# Protecting soymilk flavor and nutrients from photodegradation

**DOI:** 10.1002/fsn3.222

**Published:** 2015-03-18

**Authors:** Laurie M Bianchi, Susan E Duncan, Janet B Webster, Daryan S Johnson, Hao-Hsun Chang, Joseph E Marcy, Sean F O'Keefe

**Affiliations:** 1 Department of Human Nutrition, Foods and Exercise, Wallace Hall, Virginia Polytechnic Institute and State University (Virginia Tech)Blacksburg, Virginia, 24061; 2 Department of Food Science and Technology, Virginia Polytechnic Institute and State University (Virginia Tech)1230 Washington St. SW, Blacksburg, Virginia, 24061; 3 Fralin Life Science Institute, Virginia Polytechnic Institute and State University (Virginia Tech)101B Fralin, West Campus Drive, Blacksburg, Virginia, 24061; 4 Kraft Foods Group, Kraft Foods North America1701 West Bradley Ave., Champaign, Illinois, 61821

**Keywords:** Light, oxidation, packaging, sensory

## Abstract

Five different packaging treatments were studied over a 36-day period to determine if they protected soymilk from photo-oxidation. Soymilk was packaged in high-density polyethylene (HDPE) bottles with and without light protective additives (LPA). Two controls [(1) no LPA (translucent appearance); (2) a light-protected control (foil overwrap over no LPA control)] and three LPA-containing treatments, Low (0.6% TiO_2_), Medium (1.3% TiO_2_), High (4.3% TiO_2_) were studied. Bottles were stored in a lighted refrigerated display case (average light intensity between 800 to 2200 lux; 3°C) for 36 days and evaluated weekly. Soymilk packaged in high LPA bottles was protected from developing light-oxidized off-flavors and odors for a minimum of 15 days. High LPA bottles provided protection for riboflavin and controlled development of photooxidative products for approximately 29 days.

## Introduction

Soymilk (SM) sales have increased significantly from $250 M/year in 1996 to $1.25 B/year in 2011 (Soyfoods Association of North America 2012). The increase in sales may be attributed to improved processing methods and sensory attributes of soymilk as well as increased consumer interest in functional foods for health value. Soymilk contains heart-healthy polyunsaturated fatty acids (PUFA; 63% of total fat), including the omega-3 fatty acid linolenic acid (18:3 [n-3]), which are susceptible to autoxidation and photooxidation (Frankel 1984; Min and Boff 2002).

Photooxidation of soymilk occurs when photosensitizing molecules (e.g., riboflavin [Rb]), are activated by light energy and contribute to oxidation of PUFA. This leads to decreased health value of the product as well as development of off-flavors (Chang and Duncan 2012). Bovine milk, which is composed of approximately 31% unsaturated fatty acids (Mansson 2008), is also highly susceptible to photooxidation. Riboflavin is found naturally in bovine milk at a concentration of 1.87 *μ*g/mL and occurs in soymilk naturally at 0.7 *μ*g/mL (Gebhardt and Thomas 2002). However, riboflavin is listed as an added ingredient on soymilk food labels. Kwok et al. (2002) reported Rb in soymilk at concentrations of 2.44 mg/kg. Porphyrin structures, such as chlorophyll, also may function as photosensitizers; their presence in milk is suggested but not confirmed (Wold et al. 2005; Webster et al. 2011) nor is there any reported concentration of chlorophyll in soymilk.

Because soymilk is higher in PUFA and Rb than bovine milk, the potential for photooxidation is higher. This suggests that photooxidation, which results when singlet oxygen is produced, could rapidly decrease shelf-life of soymilk due to degradation of flavor quality. Other components of soymilk complicate this theory, however. Soymilk contains polyphenols that are also known to be antioxidants; however, under certain conditions, such as higher concentrations or interactions with other components, these polyphenols can act as prooxidants (Sisa et al. 2010). Additionally, the carotenoid vitamin A, which is enriched in bovine milk (2.08 IU/mL) and in soymilk (O.87 IU/mL), behaves as an antioxidant, but is present at different levels. Therefore, there are many factors in these two beverages that may result in different responses to light.

Soymilk and bovine milk are commonly packaged in paperboard or high-density polyethylene (HDPE) packaging. Packaging for light-sensitive products often has light protective additives (LPA), such as titanium dioxide (TiO_2_), added. LPA molecules interfere with light transmission, creating a visually opaque or colored packaging material that limits light penetration into the product. Novel approaches for modifying polymer material opacity and light interference for protecting food quality can improve product quality (Webster et al. 2009).

The purpose of this study was to determine the effect of light on the oxidative stability of soymilk and subsequent quality, as measured with sensory testing and chemical analyses. Efficacy of LPA in HDPE packaging in protecting soymilk quality was assessed.

## Materials and Methods

### Packaging

HDPE bottles (dimensions: 7.16”ht × 3.29”w × 2.1” deep; volume to overflow: 528 mL) were manufactured with three experimental TiO_2_ characteristics (DuPont™, Wilmington, DE) as well as an HDPE control with no LPA. Titanium dioxide, especially the rutile crystalline form, has the ability to attenuate various wavelengths of light. Pigmentary size rutile has the ability to attenuate light via absorption and scattering due to the particle size range (Egerton 2014). Rutile attenuation for wavelengths less than 380 nm (UV attenuation) is achieved via the absorption mechanism while the scattering phenomenon is related to the wavelengths greater than 380 but less than 700 nm (visible attenuation). Many ingredients present within fluid dairy applications have sensitivity to both UV and visible wavelengths. As such, a pigmentary size rutile, one that absorbs and scatters, is required for this evaluation.

**Figure 1:**
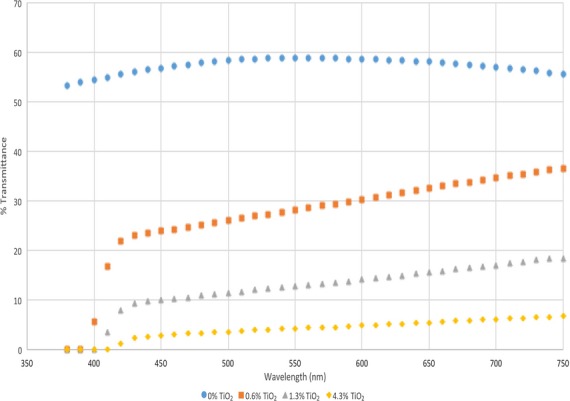
Percent transmittance spectra of the different levels of TiO_2_ added to the HDPE treatment bottles.

The three experimental treatments were differentiated by LPA contained within the bottle resin, yielding bottles with different levels of light protection. For experimental purposes, LPA-containing treatments were identified as Low (0.6% TiO_2_), Medium (Med; 1.3% TiO_2_), and High (4.3% TiO_2_), to indicate the targeted level of light protection. The transmittance spectra for each treatment bottle are shown in Fig.1. Control packaging treatments included foil-wrapped light-protected control bottles (F = foil-wrapped; 0% TiO_2_), which provided no light penetration to the product, and control bottles (no LPA, no foil overwrap; 0% TiO_2_), identified as clear (C) because the product could be readily seen through the bottle although the package was translucent. Milk in the treatment bottles was not readily visible as the bottles were opaque. Bottles and caps were sanitized with 100 ppm chlorine solution, rinsed with deionized distilled water, and drained prior to filling with soymilk product.

### Product

Soymilk (PS Lite, 128 one-half gallon paperboard containers, Kroger Co., Cincinnati, OH) was purchased from a local supermarket on the delivery date and transported immediately (5 min drive) to the Virginia Tech Food Science and Technology Department in chilled portable coolers. The product label stated the following ingredients: filtered water, whole organic soybeans, organic evaporated cane syrup, calcium carbonate, natural flavors, sea salt, carrageenan, vitamin A palmitate, vitamin D_2_, Rb, and vitamin B_12._

### Package filling and product storage

#### Package filling

Half-gallon cartons of soymilk (*n* = 128) were commingled into a clean, sanitized bucket (5 gallon GeFcontainer, General Films, Inc. Covington, OH) throughout the filling process. The product was kept covered and cold on ice throughout the filling operation. Each bottle was filled with soymilk (450 mL), using a peristaltic pump (Wheaton Unispense II, Millville, NJ) and sanitized tubing, under a positive airflow hood (Atmos Tech Industries, Ocean NJ) to minimize incidental microbial contamination from the air. Packages were immediately capped and stored in a darkened portable cooler on ice, then transferred to a refrigerated cooling system. Product was stored under refrigerated conditions, with product evaluation occurring at seven intervals over 36 days.

#### Shelf-life storage conditions for sensory testing

Filled bottles were placed into a refrigerated Friedrich Floating Air beverage case (Friedrich 60-10-1056; San Antonio, TX) to simulate retail conditions. The cooler was glass fronted with 5 doors and equipped with cool white, 32-watt fluorescent light bulbs (Alto II, Panasonic, Maple Grove, MN), simulating retail conditions. Above each of three shelves, 2 fluorescent light bulbs ran the horizontal length of the dairy case over each shelf at one-fifth and four-fifth the distance from the front of the shelf. Bottles were 11.43 cm below horizontal lights. Six light bulbs were positioned vertically at the front of the dairy case, at each end of the case and between each door junction.

Bottle placement was randomized so that all treatments were distributed randomly within the dairy case to reduce the effects of different lighting intensity within the case. Temperature and light intensity were measured routinely. The average temperature of the dairy case was 2.7°C ± 0.8. Over the 5-week storage study, light intensity measurements were taken in three general locations on each treatment for each day of analysis. Light intensity averaged 1122 lux ± 439 (range: 355–1942 lux), depending on sampling location. Because of this broad range of light intensity, 10 bottles of each treatment and 20 bottles of each control were randomly selected for sampling on each day of analyses. Product from at least two bottles per treatment was used for each analytical assay.

#### Shelf-life storage conditions for analytical testing

Chemical analyses were completed in two experimental blocks (A, B) and were separate from the sensory study. Block A was completed in April and block B was completed in August. A failure of the refrigerated dairy case to maintain temperatures under the conditions of the summer heat required a change in sample storage; this situation created the need for blocking.

Soymilk was obtained and filled into bottles as described previously. Each bottle was labeled on the bottom, filled, and randomly placed into the same dairy case as used for the sensory tests (block A) or a walk-in cooler (block B) (Tonka, Hopkins, MN). Randomization was completed to ensure that bottles from each treatment group were pulled out from random areas of the cooler on each day of analysis. In the first study (Block A), light intensity was measured over each bottle and averaged 2186 lux ± 867 with a range from 396 to 3970 lux, depending on sampling location. Temperature averaged 3.0°C ± 0.87. For the second study (Block B), temperature was taken in two different areas of the walk-in cooler. For Block B, temperatures ranged from 5 to 8°C with an average of 5.7°C ± 0.9. Light intensity was measured in five random locations above the bottles on day 1, with an average light intensity of 794.2 lux ± 43.1. Philips 34-W fluorescent light bulbs (Panasonic, Maple Grove, MN) were placed 12.7 cm over the bottles on two length sides of the area of the treatment bottles. Treatment bottles were pulled out of the dairy case/cooler on days 1, 4(block A)/3(block B), 8, 15, 22, 29, and 36. Averages of all data for both blocks are presented.

### Sensory testing

#### Sensory testing

IRB approval for use of human subjects in research was obtained from the Virginia Tech Institutional Review Board (IRB #11-477, approved Oct 14, 2011). Panelists were recruited from student, faculty, and staff at VT and the local community members. Ninety-eight panelists were targeted to complete the sensory testing for each day of evaluation to achieve targeted statistical parameters (alpha, beta, and proportion of discriminators [*p*_d_]) as described below. Panelists completed informed consent prior to initiating the sensory test. Panelists evaluated the samples in partitioned sensory booths, equipped with a touch screen monitor for data collection, under white lighting. Data were collected using Sensory Information Management Software (SIMS 2000; Sensory Computer Management, Morristown, NJ).

Triangle tests for difference as well as for similarity were completed on each day of evaluation using the unified approach to similarity and difference testing (Meilgaard et al. 2007). Testing for similarity in soymilk flavor was completed for the three LPA experimental treatments compared to the foil-wrapped light-protected (F) control (F:Low, F:Med, F:High); testing for difference was completed for all experimental treatments compared to the no LPA light-exposed (C) bottles (C:Low, C:Med, C:High). A comparison of the two controls (F:C) was also completed. Testing for similarity provided determination that the opacity of the package protected as well as light-protected control bottles. Testing for difference allowed determination that the packaging opacity protected better than light-exposed controls.

#### Sensory statistical design and parameters

An incomplete randomized block design was used, with each panelist receiving three 3-sample sets (nine total samples) on each testing date. This design reduced biases due to sensory fatigue. The order of samples within a comparison set was organized in a balanced order so that all possible combinations were presented an equal number of times. The order of the three sample sets was randomized so that there would be no bias based on the order of presentation, to reduce the influence of fatigue. Each set of three samples had two identical samples from one packaging treatment and one sample from a different treatment. The panelists were instructed to smell and taste the samples from left to right and choose the sample that was different, using the touch screen monitor. Panelists were required to choose a sample even if they could not detect a difference. Water and unsalted oyster crackers were given to each panelist for consumption to cleanse the palette between each triangle test.

Statistical parameters for the triangle test for similarity were established prior to beginning the study, comparing treatment packages to foil-wrapped controls, based on an *α* of 0.20, *β* of 0.05, and a *p*_d_ of 0.30. For triangle test for difference, comparing treatment packages to clear controls, an *α* of 0.05, a *β* of 0.20, and a *p*_d_ of 0.30 was preestablished. However, because the number of panelists participating on any given day of evaluation varied, the actual post hoc calculated parameters varied. Analysis of data was completed as described by Meilgaard et al. (2007).

### Chemical analysis

#### Analytical assessment

Chemical analyses included Rb degradation, thiobarbituric acid-reactive substances (TBARS), and GC-MS analysis of selected headspace volatiles. Analysis of bacterial contamination was completed on 3M Petrifilm™ aerobic petri film (3M, St. Paul, MN) on each day of chemical analysis. Bottles were pulled out each morning and soymilk portioned into appropriate vials for analysis.

Rb analysis was done according to a modified assay of AOAC number 960.65 in which the fluorescence of Rb was measured on a spectrofluorometer with excitation at 450 nm and emission at 520 nm (Shimadzo Scientific Instrument, Inc., Columbia, MD) (AOAC 1998).

Thiobarbituric-reactive substances (TBARS), reported as mg/kg product, were assayed in each sample. The TBARS assay is a measurement of formation of malondialdehyde, a secondary product of oxidation and an indicator of the production of other secondary volatile oxidation products. The procedure was modified from Spanier and Taylor (1991) for milk analysis.

Volatile analysis was completed on control and treatment samples. Volatile compounds from the soymilk were extracted and separated using solid phase microextraction-gas chromatography (SPME-GC). An HP 5890 GC with 5972 series mass selective detector HP5MS (Hewlett Packard, Palo Alto, CA) was used to separate and identify volatile headspace compounds. Samples (8 mL) were heated in 20-mL amber glass vials to 45°C on an RCT basic heater with an ETS-D4 Fuzzy Controller (IKA Werke, Wilmington, NC), while being stirred. An 85um carboxen-polydimethyl siloxane (PDMS) solid-phase microextraction (SPME) fiber (Supelco, Bellefonte, PA) was used to adsorb volatile compounds. The fiber was exposed to the headspace for 20 min. Volatiles were desorbed from the fiber onto an HP-5MS capillary column (30 m × 0.25 mm id × 0.25 *μ*m film thickness) to separate and analyze volatiles. The following procedure was used: Helium gas flow: 28–36 cm/sec; injector temperature: 280°C; and detector temperature: 280°C. The initial temperature of 35°C was held for 0.5 min then ramped by 15°C/min to 180°C and held for 0.5 min. The temperature then was ramped by 20°C/min to 260°C with the final temperature held for 0.5 min. The total run time was 15.17 min and the program was run in the splitless mode. The chromatograms were integrated using HP ChemStation software (Hewlett Packard, Palo Alto, CA). The identification of the compounds was made by the combination of Wiley275 Mass Spectral Library software and retention time of an authentic standard compound, hexanal (98%, Sigma Aldrich, St. Louis, MO).

#### Statistical analyses

Results of the chemical analyses of the soymilk were evaluated using ANOVA with a two factorial design and blocking for each experimental block. Main effects of package and time were the two factors. Interaction was tested as was each individual factor using *α* = 0.05 (JMP 9.0 software, SAS Institute Inc, Cary, NC). Contrasts were performed for all bottle treatments to determine failures over time compared within treatment, against foil-wrapped and clear controls on each day, and against foil-wrapped control, day 1 for each response of Rb, TBARS, and hexanal. These are described in detail in results and discussion.

## Results and Discussion

### Effectiveness of packaging in protecting sensory quality

Packaging treatments were tested for similarity of soymilk sensory quality (*β *= 0.05, *α *= 0.30, *p*_d_* *= 0.30) compared to soymilk in foil-wrapped (F) packages to test the hypothesis that LPA packaging treatments protected the milk as well as an optimal light-protected condition. The statistical parameters for similarity testing were set to reduce type II error. Difference testing was performed against the no LPA bottles (C) to test the hypothesis that LPA-packaging treatments provided better protection to oxidation than no LPA control. The statistical parameters (*α *= 0.05, *β *= 0.30, and *p*_d_* *= 0.30) for difference testing were set to reduce Type I error. A reasonable estimate for proportion of discriminators is that 30% of the population would be able to discriminate oxidized flavor and odor in soymilk; this estimate was based on previous experience in our laboratory. It also must be noted that some of the panelists consistently participated in sensory tests over the testing period where as others participated only a limited number of times.

Sensory difference testing illustrated that experimental packaging treatments protected soymilk from photooxidation related to the LPA design and the duration of light exposure (Table1).

**Table 1:** Summary table of statistical significance (*P* < 0.05) for evaluation of sensory difference and sensory similarity of soymilk for each packaging treatment (Low, Med, High)^1^ compared to controls (Clear: C; Foil-wrapped: F) over extended shelf-life lighted storage (1122 lux; 2.7°C)^2^

Package treatment comparison	Day 1	Day 4	Day 8	Day 15	Day 22	Day 36
Difference test: *α *= 0.05, *β *= 0.30, *p*_d_* *= 0.30						
C: low	0.15	0.42	0.84	0.56	0.001*	0.000*
C: med	0.78	0.84	0.34	0.008*	0.02*	0.17
C: high	0.009*	0.56	0.05	0.038*	0.004*	0.02*
F: C	0.04*	0.08	0.01*	0.0001*	0.000*	0.003*
Similarity test: *α *= 0.30, *β *= 0.05, *p*_d_* *= 0.30						
F: low	0.08	0.77	0.01*	0.03*	0.000*	0.003*
F: med	0.05	0.65	0.01*	0.56	0.000*	0.008*
F: high	0.25	0.14	0.76	0.56	0.02*	0.001*
Panelist/test	35	36–38	37–41	41–42	40–42	41–42

* *P* < 0.05.

1 Controls: F = Foil (HDPE (0% TiO_2_ with foil overwrap); C = Clear (HDPE bottles with no TiO_2_ or foil); Treatments: Low (0.6% TiO_2_), Med (1.3% TiO_2_), High (4.3% TiO_2_). F:C provides definition of how controls are differentiated within testing conditions.

2 Exposure to light (1172 lux ± 439) in hours: day 1 = 20, day 4 = 92, day 8 = 188, day 15 = 356, day 22 = 524, day 36 = 860.

As expected, there were significant differences between the control (F:C) packaging treatments within 20 h of light exposure (day 1; *P* = 0.044) and consistently (exception on day 4 (92 h); *P* = 0.075) from day 8 (188 h). This indicates that soymilk is photo-responsive to low to moderate retail lighting conditions within relatively short storage times with a resulting impact on sensory quality. Similarity testing showed the High LPA treatment was as effective as the light-protected control (F) in protecting soymilk flavor for at least 15 days, but was differentiated on day 22; however, this treatment package still afforded some protection as the soymilk was still differentiated from the soymilk packaged in the clear control during the latter part of the storage study. With the exception of day 4, the other two treatments (Low and Med LPA) were statistically different (*P* < 0.05) by day 8 from light-protected control (F), indicating that they did not protect from off-flavors or aromas as well as full light protection and were no more effective than the light-exposed no LPA (C) packaging for the first two weeks. However, soymilk packaged in Low and Med LPA treatments did not change in flavor as extensively as product packaged in the clear control, as there were product differences at days 15 (Med) and 22 (Low). Soymilk within the clear (no LPA) packaging had significant photooxidation occurring within these bottles that exceeded that of the product in the Low and Med LPA packaging.

The lack of sensory difference between the two controls (F:C) on day 4 appears to be an anomaly, which may be partially explained by the participants on that day of evaluation. When the portion of discriminators was assessed for each sensory day, the number of discriminators was lower on day 4 of sensory testing, indicating that not as many panelists were able to discriminate the oxidized aroma/flavor of the soy milk. The challenge is to determine whether that is because there truly was no difference among the products or whether the portion of the population that completed the test on that day were less sensitive (than the population on day 1, for example). Although many participants routinely participated throughout the study, there was no way to get 100% compliance in participation so there were some changes in the participant pool on each date.

While changes in product quality were obvious over time, we also acknowledge that participants also learned from experience. We are 95% confident that the true population that can distinguish differences is lower at the beginning of the study, when sensory differences are relatively small, and increases with time as product quality changes with light exposure. We also acknowledge, however, that inadvertent “training” of the panelists may also occur over time. As many of the panelists returned throughout the study, they may have become more adept at detecting differences. There also appears to be a pattern in which the true populations that can distinguish between the foil-wrapped and clear control treatments is higher than the other comparisons, as there would be greater difference in sensory quality between products in these two controls.

### Effectiveness of packaging at protecting riboflavin and limiting oxidative degradation

The chemical analyses of the evaluation were treated as blocks because there were differences in experimental conditions and to reduce effects associated with greater light intensity and variation in the first study (block A) compared to the second study (block B). Nonetheless, each block demonstrated similar trends. Furthermore, statistical analysis of block effects indicated that each block did not differ from one another significantly (Table2). Power was determined for each block and each had a power estimate of 1, demonstrating that the blocks can stand independently. This indicates that the treatment packages are effective for the lower, less variable light intensity (block B) as well as under conditions of higher light intensity and greater variability in intensity (block A). Both block A and B showed that the High LPA treatment provided better protection for Rb degradation and photooxidation products (TBARS, hexanal). Results shown are the average of all data from both blocks. However, because the lighting conditions of block B were dissimilar from the lighting conditions of the sensory experiment, comparisons between sensory analysis and chemical analysis were only discussed regarding block A.

**Table 2:** ANOVA table for effects test for response of Rb concentration testing for package, time (days), block, and interactions (*α *= 0.05)

Source	df	Sum of squares	*F* ratio	Probability > *F*
Package	4	3.460	36.682	<0.0001*
Block	1	0.057	2.427	0.12
Day	6	4.081	28.839	<0.0001*
Package*Block	4	0.296	3.136	0.02*
Package*Day	24	2.033	3.592	<0.0001*
Block*Day	6	0.553	3.907	0.002*
Package*Block*Day	24	0.940	1.660	0.05

* *P*-value < 0.05 indicates statistical significant difference.

df = degrees of freedom; *F* = Variance of the group means/mean of the within group variances.

**Figure 2:**
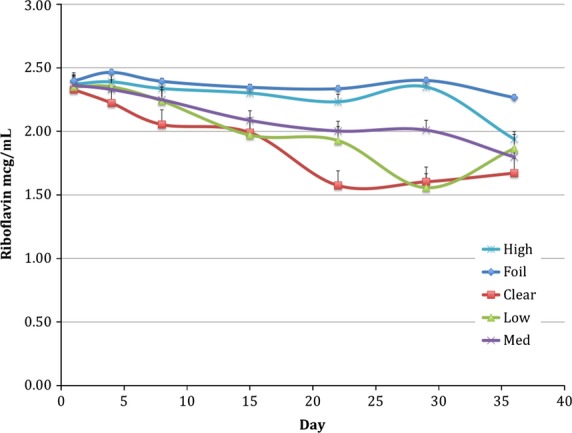
Rb degradation in packaging treatments, Low (0.6% TiO_2_), Med (1.3% TiO_2_), and High (4.3% TiO_2_), compared to foil-wrapped and clear packaging over a 36-day period.^1^ Average of data in both Block A and Block B. ^1^Exposure to light in hours: day 1 = 23.5, day 4 = 84, day 8 = 191, day 15 = 359, day 22 = 527, day 29 = 696, day 36 = 864.

Rb concentration in fresh soymilk was 2.33 *μ*g/mL. This is in agreement with Kwok et al. (1998) who found riboflavin to be present in soymilk at a concentration of 2.44 mg/kg. In soymilk packaged in foil-wrapped control bottles, Rb remained relatively unchanged over time, with only 5.4% loss of Rb over the 5-week period (Fig.2). The effect of packaging and storage time (“day”) were statistically significant (*P* < 0.05) as were the two-way interactions (package*block, package*day, and block*day). In the clear (no LPA), Low, and Med LPA packaging treatments, the degradation of Rb was greater than in foil-wrapped bottles, with losses of 28.3%, 20.9%, and 23.7%, respectively, over the 5-week storage. High LPA packaging treatment provided optimal protection up to day 29 as the Rb concentration was not significantly different from the light-protected control (F) up to that time, with only 0.8% loss of Rb. However, degradation in soymilk in the High LPA packaging did occur from day 29 to day 36, with a 5.4% loss of Rb in the light-protected soymilk (F) and an 18.1% decrease in the High LPA-packaged soymilk.

Munoz et al. (1994) studied percent of riboflavin loss in dairy milk over a 6-day storage period and found that riboflavin suffered losses of 16% to 23.4% after only 6 days in dairy milk. In a previous study in our laboratory (Johnson et al. 2015), using the same packaging and storage conditions as our soymilk study, dairy milk (2% milkfat) decreased in Rb concentration by approximately 5% in foil-wrapped HDPE and 16% in high LPA packages at day 8; Rb losses were approximately 45% in the clear package. Our soymilk study shows losses of riboflavin of 14.3% in product in clear packages and only 2.4% in the high LPA packages at day 8. It should be noted that Munoz et al. (1994) stored the milk in open packages and our packages were sealed.

Statistical contrasts that compared the Rb concentration of the light-protected soymilk (F) from day 1 to the concentration on each subsequent day of analysis and all of the other bottles on all days were completed. As expected, a contrast completed between foil-wrapped control, day 1 (F1) versus other TiO_2_-packages demonstrated significant differences. However, comparison of the Rb concentration in F1 indicated no differences compared to High LPA bottles. There were no differences in Rb concentration among any of the packaging treatments at the beginning of the study. Differences in Rb concentration in High LPA-packaged soymilk were not evident compared to F1 control until day 36.

**Figure 3:**
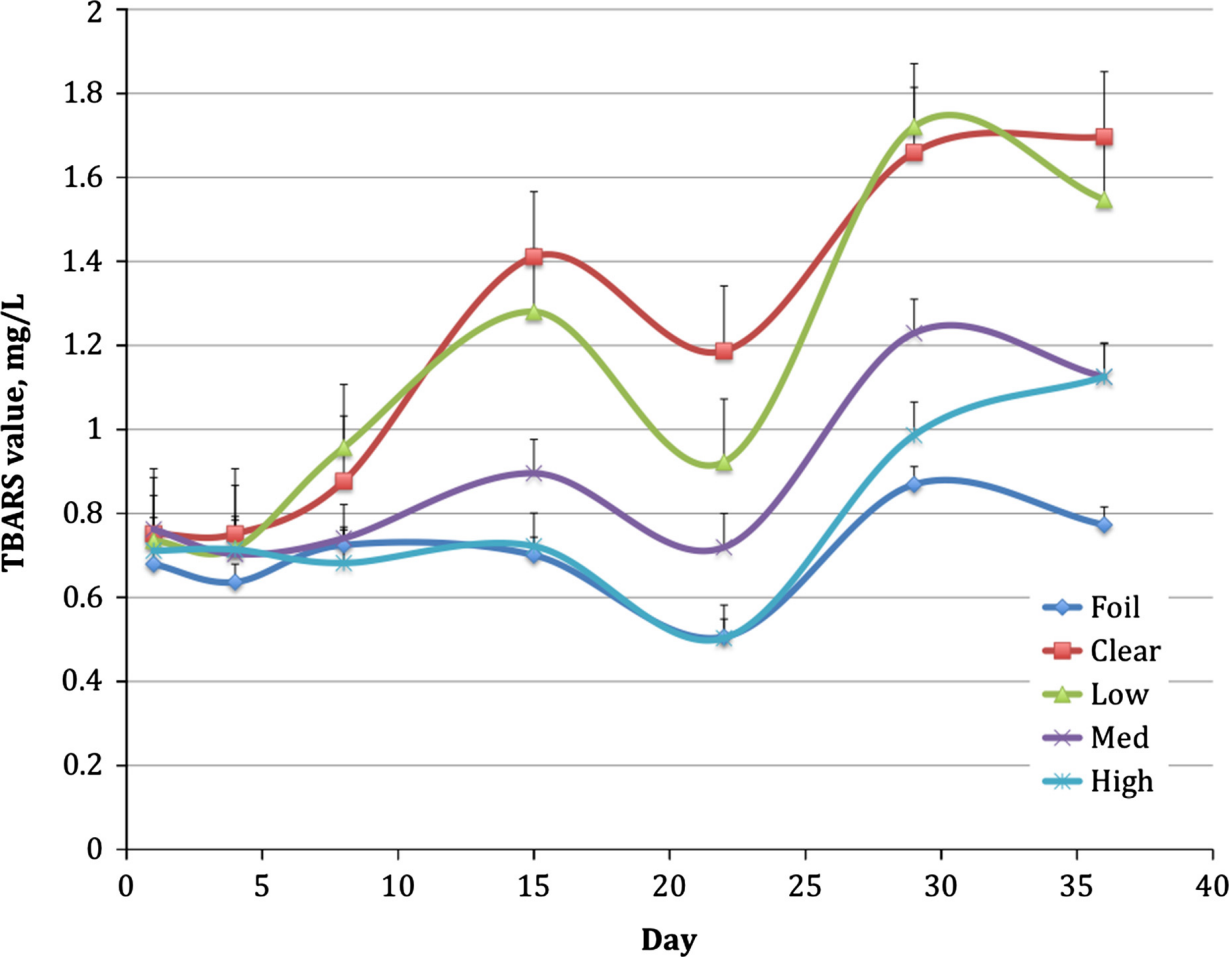
TBARS values in packaging treatments, Low (0.6% TiO_2_), Med (1.3% TiO_2_), and High (4.3% TiO_2_), compared to foil-wrapped and clear packaging over a 36-day period.^1^ Average of data in both block A and B. ^1^Exposure to light in hours: day 1 = 23.5, day 4 = 84, day 8 = 191, day 15 = 359, day 22 = 527, day 29 = 696, day 36 = 864

**Figure 4:**
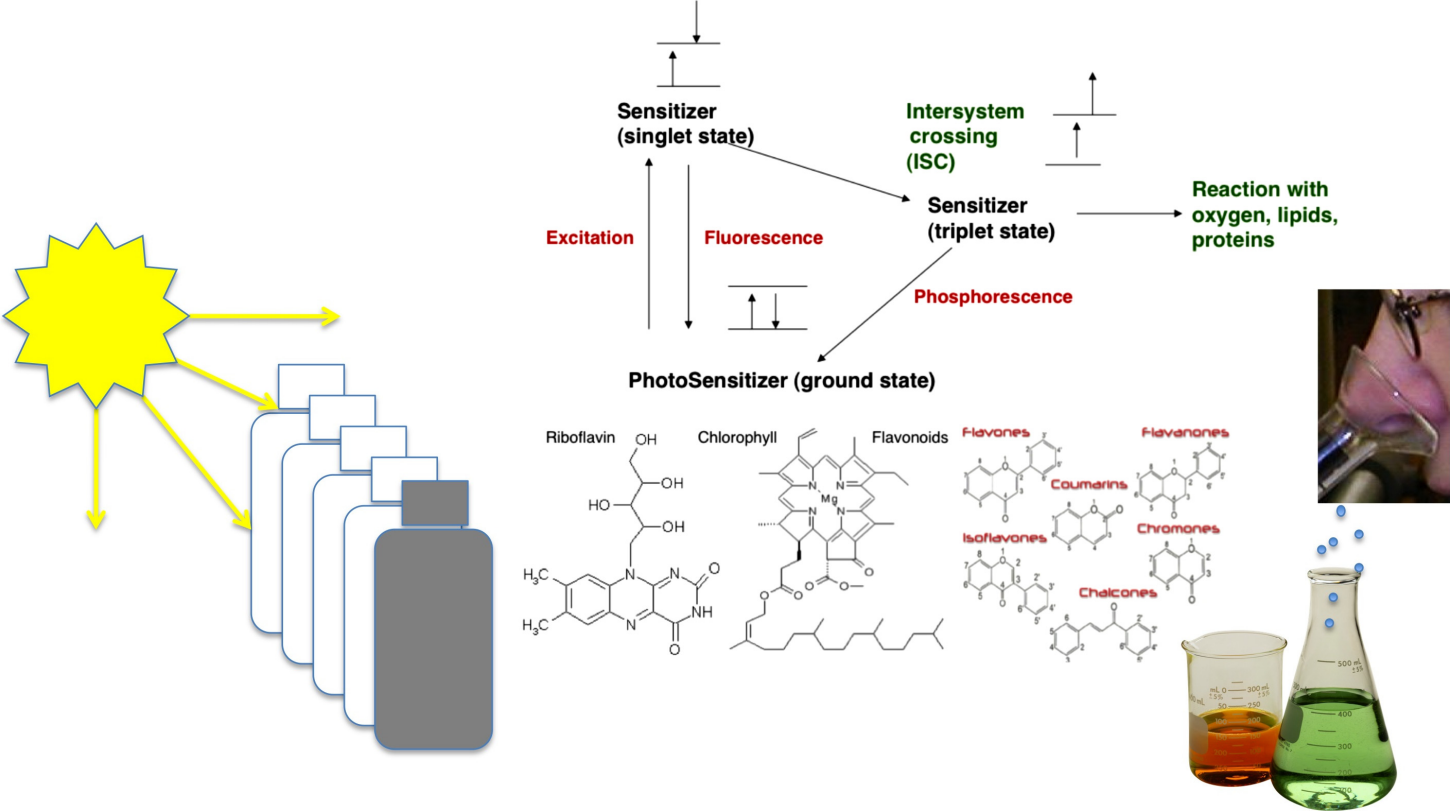
Integration of polymer chemistry, photochemistry, volatile chemistry, and sensory analyses provides packaging design guidance.

We also measured oxidation using TBARS values as an indication of aldehydes, which are secondary products of photooxidation. This method has been studied extensively in meat and milk oxidation and been shown to increase in concentration as photooxidation occurs. It has also been shown to correlate with changes in sensory characteristics of rancidity (Wang et al. 2001; van Aardt et al. 2005; Johnson et al. 2015). TBARS values for both block A and B of the study increased over time, as shown in Fig.3. As expected, foil-wrapped packages protected the soymilk optimally as TBARS was lowest in soymilk from this packaging treatment over time; significant differences did not occur until day 36. Soymilk in High LPA packages had low-TBARS values, mimicking TBARS of soymilk in foil-wrapped bottles up to day 29. However, between day 29 and 36, TBARS values increased in soymilk in High LPA packages, following the trend observed for Rb, and still significantly lower than clear packages. Differences were noted in TBARS values at day 29 (Table3). In a previous study in our laboratory on fluid milk (Johnson et al. 2015), sensory detection between control and packaging treatments were frequently associated with TBARS levels of 1.3 mg/L in the product. TBARS values in the soymilk were 1.3 mg/L on day 15 in soymilk in Low and 0.9 mg/L in Med LPA bottles and this is the point at which sensory differences were first detected. Although Rb degradation was not significantly different at day 29, the secondary products of photooxidation increased in High LPA bottles compared to foil-wrapped day 1 concentrations. Other components of soymilk, such as protein, may be undergoing oxidation (Karel et al. 1975), or chlorophyll, another known photosensitizer, may be contributing to additional photooxidation pathways of components in the soymilk. Measuring chlorophyll in complex beverage systems is challenging. Mendiola et al. (2008) attempted to determine chlorophyll concentration in a mixture of orange juice and soymilk; however, they could not detect it under their specific HPLC conditions. Wold et al. (2005) suggests that chlorins in dairy-based cheese contributed to photooxidized off-flavors based on fluorescence spectroscopy. Intawiwat et al. (2010), using fluorescence spectroscopy, identified very small peaks in the 630 to 680 nm range in bovine milk as protoporphyrins and chlorophyllic compounds. We did not measure chlorophyll in soymilk for this study but determining if or how chlorophyll contributes to off-flavors associated with photooxidized soymilk is important for optimizing packaging for protecting soymilk flavor and nutrient quality. Additionally, soymilk contains a complex mixture of antioxidants including isoflavones (Gebhardt and Thomas 2002; Chiarello et al. 2006). These antioxidants may also play a role in the inhibition of photooxidation, which may help explain why the comparable studies of dairy milk (Johnson et al. 2015) and soymilk showed differences in Rb concentration over the shelf-life. Antioxidant compounds in soymilk, however, may also be sensitive to photooxidation after some degree of light exposure. In soymilk, how all these photosensitizers and antioxidants should best be protected from light using packaging is a relevant question. Integrating the understanding of polymer chemistry, photochemistry, oxidation chemistry, volatile chemistry, and sensory evaluation is valuable in developing packaging solutions (Fig.4).

**Table 3:** ANOVA table for effects test for response of TBARS values testing for package, time (days), and block and interactions (*α *= 0.05)

Source	df	Sum of squares	*F* ratio	Probability > *F*
Package	4	4.906	124.750	<0.0001*
Block	1	0.024	2.393	0.1264
Day	6	8.324	141.104	<0.0001*
Package*Block	4	0.504	12.821	<0.0001*
Package*Day	24	2.743	11.626	<0.0001*
Block*Day	6	2.076	35.194	<0.0001*
Package*Block*Day	24	0.985	4.175	<0.0001*

* *P*-value < 0.05 indicates statistical significant difference.

df = degrees of freedom; *F* = Variance of the group means/mean of the within group variances.

There is a dip in TBARS at day 22. This trend has been seen before (Wang et al. 2001). These authors studied characteristics of flavored soymilk over time and demonstrated a decrease in TBARS values in refrigerated soymilk by day 14 and also at day 28. They hypothesize that the decrease is due to secondary photooxidation reaction products binding to proteins in the soymilk. They further postulate that this binding may be the formation of Schiff bases due to Strecker degradation in which carbonyls formed from oxidation are reacting with amine groups in the protein (Damodaran 2008). We saw a subsequent increase in TBARS values following the decrease at day 22. Wang et al. (2001) corrected for possible sugar oxidation that may occur from the sucrose in the soymilk, whereas we did not. Perhaps the subsequent increase in TBARS values is due to oxidation of proteins (Karel et al. 1975).

TBARS values that coincide with the sensory changes in soymilk are lower than what have been seen in similar studies with cow milk. Our hypothesis is that there are other changes in the soymilk that are occurring that are contributing to sensory changes, which will be discussed in more detail below.

**Figure 5:**
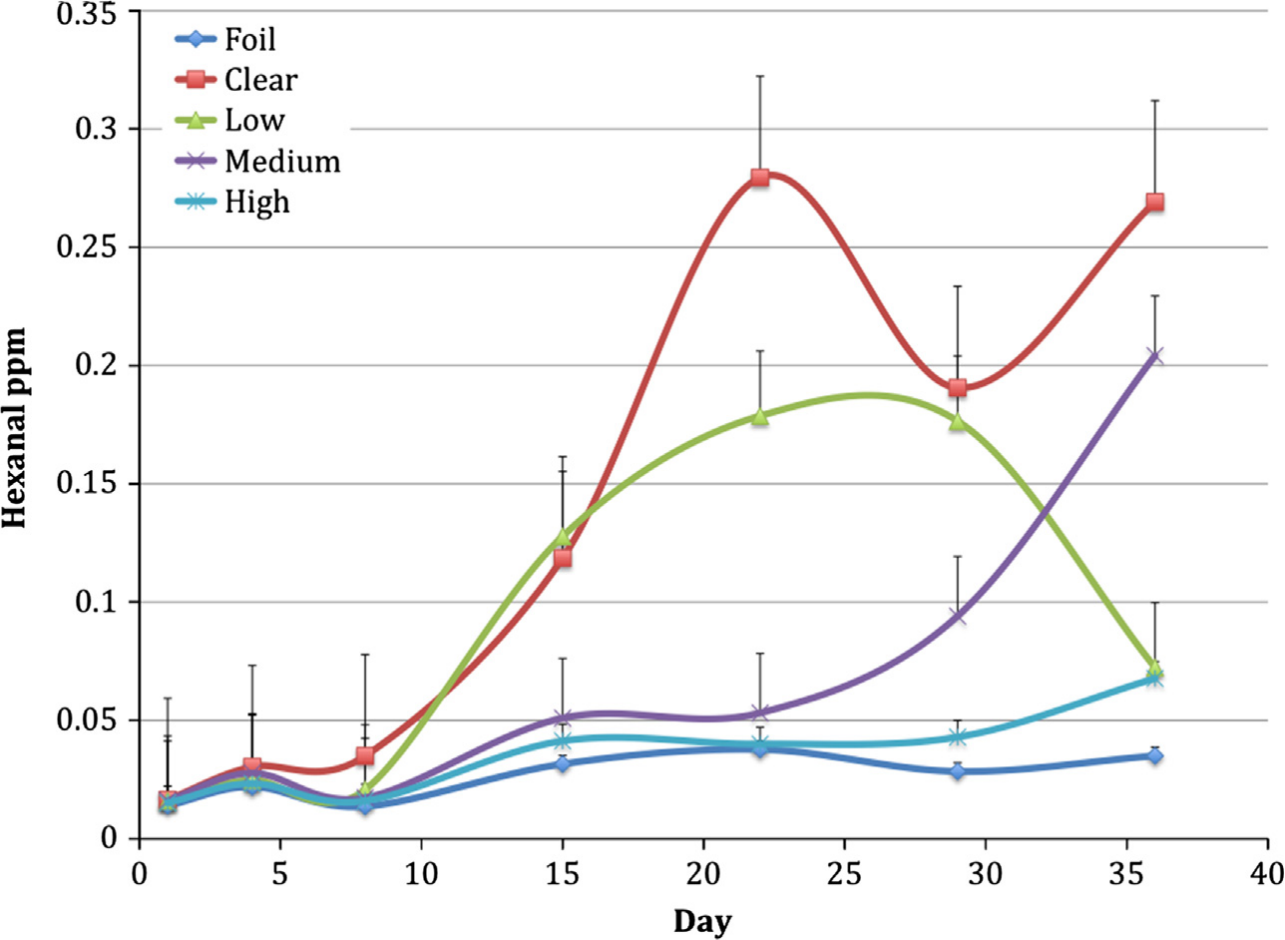
Hexanal concentration in packaging treatments, Low (0.6% TiO_2_), Med (1.3% TiO_2_), and High (4.3% TiO_2_), compared to foil-wrapped and clear packaging over a 36-day period.^1^ Average of data in both block A and B. ^1^Exposure to light in hours: day 1 = 23.5, day 4 = 84, day 8 = 191, day 15 = 359, day 22 = 527, day 29 = 696, day 36 = 864.

Hexanal is also a known secondary oxidative product of photooxidation. Hexanal concentration of soymilk packaged in different bottles is shown in Fig.5. As expected, the high LPA treatment protected similarly to foil-wrapped bottles up to day 29 and even at day 36 protected significantly better than clear bottles. There is a wider variation of hexanal concentration in the packaging treatments over the study period. This may be explained in part because SPME analysis can provide a high degree of variability and we did not use internal standards in our analysis to account for this. Also, hexanal is a secondary oxidative product and it may be produced in a more variable manner due to possible competitive pathways. Schaich (2012) suggests that there may be alternative reactions that compete with the traditional depiction of hydrogen abstraction during the propagation stage of lipid oxidation. If other side reactions do indeed compete with hydrogen abstractions during propagation, there could be a whole myriad of products present that affect traditional measurements of oxidation, such as hexanal. These possible alternative reactions of photooxidation may be nonvolatile compounds that are not detected by GC-MS (Schaich 2012).

The ANOVA table for interactions and effects of hexanal are shown in Table4. These statistics are not blocked. However, once again, the results are an indication that packaging treatment and time do have an effect on hexanal concentration and that there is an interaction of packaging and time.

**Table 4:** ANOVA table for effects tests for response of hexanal concentration testing for package, time (days), block, and interactions (*α *= 0.05)

Source	df	Sum of squares	*F* ratio	Probability > *F*
Package	4	0.180	40.567	<0.0001*
Day	6	0.246	36.949	<0.0001*
Package*Day	24	0.179	6.729	<0.0001*

* *P*-value < 0.05 indicates statistical significant difference.

df = degrees of freedom; *F* = Variance of the group means/mean of the within group variances.

Contrasts for hexanal are similar to the results of the Rb analysis. Hexanal concentration in soymilk in different packaging treatments as early as day 1 is not significantly different from one another; over time, foil-wrapped and High LPA bottles give protection for well over a 4-week period. However, by day 36, soymilk in High LPA bottles had increased hexanal production compared to foil-wrapped bottles. The clear, Low LPA, and Med LPA TiO_2_ bottles always demonstrated higher hexanal levels than the foil wrapped bottles; thus they appear to be unable to prohibit lipid photooxidation.

While the chemical analyses are all in agreement regarding how long the high LPA package can maintain protection similar to the foil-wrapped packaging, the sensory testing indicates that High LPA treatment was not able to maintain protection. Off flavors and aromas were detected by panelists after 15 days. Possible explanations for this include the fact that human sensory is much more sensitive than chemical analysis. Perhaps human sensory testing is detecting compounds that we have not yet evaluated or are simply are not yet in high enough concentrations to be detected analytically. Certainly there are other secondary products of photooxidation that we have not analyzed, including 2-pentyl furan and 2- pentenyl furan, both of which are known oxidative products that contribute to soy oil reversion flavor (Ho et al. 1978; Frankel 1984). Furthermore, single compounds in and of themselves do not necessarily cause sensory responses, so it may be that a combination of compounds caused detection of rancidity earlier than the chemical assays.

Another explanation is that the soymilk package indicates that natural flavoring is added. It may be that a natural flavoring is oxidized or undergoing time degradation. Perhaps the degradation of the added natural flavoring is what panelists were able to detect as a difference rather than an increase in oxidized off flavors and odors attributed to soymilk components.

Rb decreased and TBARS values and hexanal increased in the foil-wrapped bottles over time, but never to the point of significant differences from the Foil day 1 control. In the High LPA bottles, the Rb decreased and TBARS values and hexanal increased over time, such that the High LPA bottles by day 36 were significantly different from initial concentrations. However, the High LPA bottles were able to protect as well as the foil wrapped bottles for at least 29 days.

High LPA packages protected soymilk from photooxidation as effectively as foil-wrapped bottles for at least 15 days in terms of human detection of off flavors and aromas. This does not necessarily mean that the High LPA package is only capable of protecting for 15 days. Normally consumers are not handling soymilk in their homes and comparing to an optimal product for each use. The sensory test conducted did not assess acceptability of the products over time, so significant sensory differences do not necessarily translate to poor quality.

## Conclusions

HDPE packaging with 4.3% TiO_2_ is able to protect soymilk from sensory changes and riboflavin degradation, which are attributed to continuous light exposure for 15 and 29 days, respectively. Other compounds in addition to hexanal and TBARS should be assessed for chemical changes in soymilk when studying oxidative contributions to sensory changes.
